# Optical coherence tomography acquisition efficiency: comparison of Heidelberg spectralis OCT2 module & Zeiss Cirrus HD-OCT model 6000

**DOI:** 10.3389/fopht.2026.1828559

**Published:** 2026-07-20

**Authors:** Charissa H. Tan, Michael S. Jensen, Shannon Fitch, Richard Ordonez, Abigail Jacketta, Jeff Courtright, Jonathan Shepherd, Mathew Vierkant, Paul Crown, Ben J. Brintz, Susan Chortkoff, Brian C. Stagg

**Affiliations:** 1 Department of Ophthalmology & Visual Sciences, University of Utah, John Moran Eye Center, Salt Lake, UT, United States; 2 Department of Ophthalmology, University of Texas Health Science Center at San Antonio, San Antiono, TX, United States; 3 School of Medicine, University of Utah, Salt Lake, UT, United States; 4 Campbell University School of Osteopathic Medicine, Lillington, NC, United States; 5 Division of Epidemiology, Department of Internal Medicine, University of Utah, Salt Lake, UT, United States

**Keywords:** Cirrus OCT, glaucoma imaging, imaging time, optical coherance tomography, Spectralis OCT, workflow efficiency, workflow optimization

## Abstract

**Aims:**

To compare optical coherence tomography (OCT) acquisition times between the Heidelberg SPECTRALIS OCT2^®^ module (Spectralis OCT2) and Zeiss CIRRUS™ HD-OCT Model 6000 (Cirrus 6000) in a glaucoma clinic setting.

**Methods:**

A prospective observational study of 37 glaucoma or glaucoma suspect patients (72 eyes) aged ≥50 years undergoing routine imaging. Each participant received OCT imaging on both platforms during the same visit, with device order randomized. Acquisition times for optic disc/retinal nerve fiber layer (RNFL) and macular scans were recorded using a standardized protocol. Subgroup analyses were performed for eyes with vision worse than 20/40. Image quality and repeat attempts were documented.

**Results:**

Total scan time was significantly faster by 41 seconds per person using Cirrus 6000 compared to Spectralis OCT2 (115.9 ± 44.7 vs. 157 ± 46.4 seconds; p < 0.001). Cirrus 6000 also demonstrated significantly shorter RNFL acquisition times (50.8 ± 29.7) compared to Spectralis OCT2 (99.5 ± 32.7 seconds; p < 0.001). Macular scan times did not differ significantly (p = 0.180). Spectralis OCT2 achieved higher completion rates and required fewer repeat attempts. Image quality scores met manufacturer thresholds for acceptable scans in most cases, though direct cross-device comparison is precluded by different scoring scales.

**Conclusions:**

Cirrus 6000 offers meaningful time savings for RNFL imaging, which may improve workflow efficiency in high volume glaucoma clinics. However, Spectralis OCT2 demonstrated greater reliability in challenging imaging conditions. Device selection should be individualized based on patient characteristics, clinic workflow, and imaging priorities. There is no single superior platform; aligning device capabilities with clinical needs can optimize both efficiency and diagnostic reliability in glaucoma care.

**Precis:**

The Cirrus 6000 achieved significantly faster OCT acquisition times while the Spectralis OCT2 demonstrated greater reliability in challenging imaging conditions. These differences highlight how platform selection can influence workflow efficiency and imaging success in glaucoma clinics.

## Introduction

Optical coherence tomography (OCT) is essential for contemporary glaucoma care and is performed routinely, from baseline evaluation to follow up monitoring. High resolution imaging of the optic disc, retinal nerve fiber layer (RNFL), and macula from OCT provides objective and reproducible structural information that complements functional testing (1, 2). These measurements play a critical role in both diagnosis and longitudinal surveillance, enabling clinicians to detect progression earlier and manage disease more effectively (3–6). As clinical volumes increase and imaging technologies evolve, the efficiency of OCT acquisition has emerged as a key operational factor in delivering timely and effective care.

Acquisition time and the associated clinical resources, including staff time, technician expertise, and training, represent non-negligible inputs into the overall flow and efficiency of a healthcare clinic (7). OCT is a standard-of-care assessment for glaucoma that allows for more standardized documentation than magnified stereoscopic visualization, with many patients undergoing at least one scan annually. In a clinic setting, dozens of patients may require OCT imaging on a single day, adding to the growing list of time-intensive tasks in busy clinics that includes patient workups, vision checks, intraocular pressure measurements, visual fields, and insurance coordination. Even modest differences in imaging time per patient can accumulate, affecting patient wait times, staff workload, and overall clinic throughput. These operational impacts may contribute to patient dissatisfaction, staff burnout, and inefficiencies that affect the entire clinical workflow.

Among commercially available OCT platforms in clinical glaucoma practice, the Heidelberg SPECTRALIS OCT2^®^ module (Spectralis OCT2, Heidelberg Engineering Inc., Heidelberg, Germany) and the Zeiss CIRRUS™ HD-OCT Model 6000 (Cirrus 6000, Carl Zeiss Meditec, Dublin, CA) are two of the most established. Although there are many OCT platforms, Cirrus 4000/5000/6000 remains the most widely used platform in the United States by installed base, while Spectralis OCT1/OCT2 is frequently used in research settings and academic practices (8). Both systems are FDA-cleared, non-invasive, and capable of acquiring high quality images suitable for glaucoma diagnosis and follow up (9). However, differences in their acquisition protocols, user interfaces, and automated reporting workflows may influence total imaging time. While prior studies have compared the diagnostic performance and structural measurements of these devices, there is limited objective data evaluating their relative efficiency in real-world clinical settings (10–14). Understanding these differences in time is particularly relevant for practices evaluating workflow optimization or considering device integration for research and clinical care.

The goal of this study is to evaluate and compare OCT image acquisition time between the Spectralis OCT2 and the Cirrus 6000 in patients with varying stages of glaucoma undergoing standard clinical imaging. Because OCT image acquisition time affects patients, clinic staff, and clinicians alike, a comparison between these two platforms may provide valuable data to inform workflow optimization, equipment selection, and resource allocation in glaucoma clinics.

## Methods

### Study population

All study participants were from glaucoma clinics at the University of Utah Moran Eye Center. Patients who were scheduled to undergo OCT imaging to monitor or evaluate glaucoma as part of their routine care were recruited to participate in the study. Included patients were 50 years of age or older and had a diagnosis of suspected, mild, moderate, or severe glaucoma in at least one eye. Patients who were unable to complete OCT testing, could not follow study directions, had active eye infections, or declined to participate were excluded. Eligible patients were identified through review of electronic medical records by the principal investigator or a sub-investigator to verify that inclusion and exclusion criteria were met. Written consent was obtained prior to enrollment. All procedures were approved by the University of Utah Institutional Review Board effective July 16, 2024 (reference number: IRB_00177814) and performed in compliance with relevant laws and institutional guidelines.

### Testing procedures

This study compared image acquisition times between two standard-of-care OCT platforms: Spectralis OCT2 and Cirrus 6000. Both devices are non-invasive and pose no physical risk to participants. Each participant underwent imaging with both Spectralis OCT2 and Cirrus 6000 during the same clinic visit, and the order of device use was randomized for each patient to mitigate effects of testing anxiety or fatigue. Randomization occurred by computer generated sequence, and the assigned order was documented.

The Spectralis OCT2 and Cirrus 6000 devices were located in the same examination room, and imaging was performed consecutively on both platforms during a single visit. For binocular patients, both eyes were imaged sequentially on one device before transitioning to the other, with the right eye always imaged before the left. The interval between devices was limited to patient data entry, which required approximately one minute. All imaging for each platform was performed by a dedicated certified study technician, with one technician assigned exclusively to the Spectralis OCT2 and a second technician completing all scans on the Cirrus 6000. Both technicians had more than five years of experience with their respective platforms, inclusive of older versions.

To assess macular thickness, Posterior Pole (Spectralis OCT2) and Macular Cube (Cirrus 6000) scans were obtained. For RNFL evaluation, Bruch’s Membrane Opening (BMO) (Spectralis OCT2) and Optic Disc Cube (Cirrus 6000) scans were performed. Automatic Real-Time (ART) was enabled for Spectralis OCT2; the mean settings were 100 frames for RNFL and 9 frames for macula. All scans were done as if it was the patients’ first time receiving the scan, so anatomic markers from prior scans were not used and manual centration was required for both devices. Per technician protocol for all new patients, scan order was fixed. Macular scans were always conducted prior to RNFL scans on the Cirrus 6000 while RNFL scans were always conducted prior to macular scans on the Spectralis OCT2. A separate study observer recorded the image acquisition time for each scan using a standardized protocol to limit discrepancies between the platforms.

Timing began when the patient placed their chin on the device and ended once an acceptable image was captured. Timing included manual centration but did not include patient data entry. If a scan was repeated due to poor image quality, the timer would restart for the repeated portion. The imaging technicians remained masked to the timing data and to the study hypothesis throughout the study. Low quality images could be repeated up to two times. Pupil dilation status and the number of prior OCT examinations performed on each platform were recorded for all participants.

### Statistical analysis

The image acquisition times were compared between the Spectralis OCT2 and Cirrus 6000 using paired t-tests. Each patient contributed one paired set of measurements (i.e., total time for both eyes for Cirrus 6000 and total time for both eyes for Spectralis OCT2). The paired t-test on the within-patient differences accounted for the correlation between fellow eyes through the matched-pairs design. Sample size calculations were based on an estimated mean acquisition time of 120 ± 30 seconds for the Spectralis OCT2. To detect a 10% reduction in time with 80% power, 36 participants were required for both the optic disc/RNFL and macular scans. Exploratory subgroup analyses were conducted according to visual acuity and analyzed using Wilcoxon signed-rank test due to small sample sizes.

## Results

A total of 37 patients contributed paired OCT measurements (**Table 1**). Participants were 73.8 ± 8.8 years old on average (range: 52–86 years). Except for two patients who had ocular prostheses in one eye, all participants underwent imaging of both eyes. Prior OCT exposure was common with Spectralis OCT2 (median per patient: 7 scans) and less so with Stratus, Cirrus 6000, and Optovue (median per patient: 0 scans). Most eyes had a visual acuity of at least 20/40, a diagnosis of primary open-angle glaucoma, and an average visual field mean deviation of -4.75 ± 7.32 dB (**Table 2**). Fifteen patients were excluded from the study. One patient withdrew consent. Fourteen (mean age: 74.9 ± 5.4) were unable to complete the scans due to lack of view from factors such as poor dilation, poor visual acuity, head tremors, cognitive abilities, or dense cataracts. Like patients who were able to complete OCT imaging, most eyes had a visual acuity of at least 20/40, however, their average mean deviation was -7.83 ± 6.12 dB.

**Table 1:** Patient demographics, pupil status, and prior OCT exposure.

Characteristic	Included (N = 37)	Excluded (N = 14)
Age (years)	73.8 ± 8.8 (range 52-86)	74.9 ± 5.4 (range 60-80)
Sex		
Female	27 (73.0%)	8 (57.1%)
Male	10 (27.0%)	6 (42.9%)
Dilated		
No	14 (37.8%)	11 (78.6%)
Yes	23 (62.2%)	3 (21.4%)
Prior OCT exposure		
Cirrus	5 (13.5%)	0 (0)
Spectralis	36 (97.3%)	12 (85.7%)
Stratus	8 (21.6%)	1 (7.1%)
Optovue	1 (2.7%)	0 (0%)
None	0 (0%)	1 (7.1%)

**Table 2:** Ocular characteristics.

Characteristic	Included (N = 72)	Excluded (N = 28)
Visual acuity (per eye)		
≥ 20/40	64 (88.9%)	21 (75%)
< 20/40	8 (11.1%)	7 (25%)
Visual field mean deviation (dB)		
OD	−4.73 ± 6.93 (n = 34)	−8.27 ± 8.33
OS	−4.78 ± 7.82 (n = 33)	−7.40 ± 8.37
OU	−4.75 ± 7.32 (n = 67)	−7.83 ± 6.12
Glaucoma type		
Primary Open Angle Glaucoma	34 (47.2%)	14 (50%)
Glaucoma Suspect	19 (26.4%)	2 (7.1%)
Narrow Angle Glaucoma	4 (5.6%)	2 (7.1%)
Pseudoexfoliation Glaucoma	4 (5.6%)	0 (0%)
Uveitic Glaucoma	2 (2.8%)	0 (0%)
Low Tension Glaucoma	2 (2.8%)	3 (10.7%)
Secondary Open Angle Glaucoma	0 (0%)	4 (14.3%)
No Glaucoma Diagnosis	7 (9.7%)	3 (10.7%)
Glaucoma severity		
Mild	17 (23.6%)	8 (28.6%)
Moderate	9 (12.5%)	7 (25%)
Severe	20 (27.8%)	8 (28.6)
Suspect	19 (26.4%)	2 (7.1%)
No Glaucoma Diagnosis	7 (9.7%)	3 (10.7%)
Other ocular conditions*		
Cataract	22 (30.6%)	
Corneal Scar	3 (4.2%)	
Corneal Dystrophy	2 (2.8%)	
Dry Eye	22 (30.6%)	
Epiretinal membrane	3 (4.2%)	
Macular Degeneration	8 (11.1%)	
Posterior Capsular Opacification	5 (6.9%)	
Pseudophakic	31 (43.1%)	
Other	23 (31.9%)	

*Comorbidity categories are not mutually exclusive. A single eye can contribute to multiple categories, so the counts exceed n = 72.

**Figure 1:**
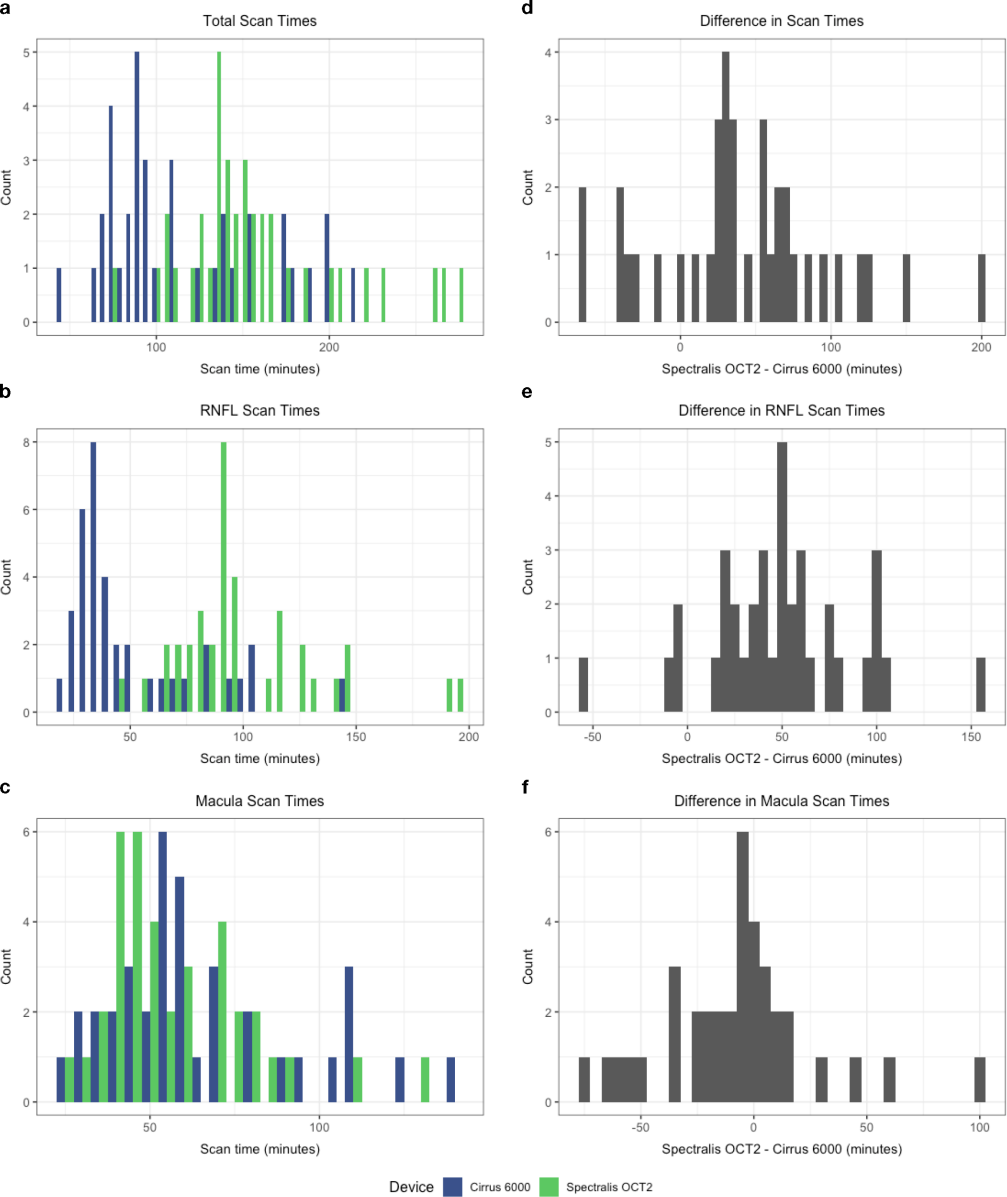
Histograms of **(a)** total scan time, **(b)** RNFL scan time, **(c)** macula scan time, **(d)** difference in total scan time, **(e)** difference in RNFL scan time, and **(f)** difference in macula scan time.

Total scan time, which includes both optic disc/RNFL and macula imaging, takes longer with Spectralis OCT2 than Cirrus 6000 (**Table 3**; **Figure 1**). Spectralis OCT2 scans took an average time of 157.2 ± 46.4 seconds while Cirrus 6000 took 115.9 ± 44.7 seconds, with a mean paired difference of 41.2 (95% CI, 22.2-60.3; p < 0.001; Cohen’s dz = 0.72). RNFL scan time was also significantly longer on Spectralis OCT2 (99.5 ± 32.7 seconds) than on Cirrus (50.8 ± 29.7 seconds), yielding a mean difference of 48.7 (95% CI, 35.8-61.6; p < 0.001; dz = 1.26). The devices took a similar amount of time to complete the macular OCT scans with a mean difference of 7.4 seconds (95% CI, -18.5-3.6; p = 0.18). In the exploratory subgroup of five patients with at least one eye with a visual acuity worse than 20/40, Spectralis OCT2 mean scan times were longer for total and RNFL scans but shorter for macular imaging. All differences were not significant (**Table 4**; p > 0.18).

**Table 3:** Scan time comparisons.

Measurement	Cirrus 6000 mean (SD)	Spectralis OCT2 mean (SD)	Mean difference (95% CI)	Cohen’s dz	P-value
Total	115.9 (44.7)	157.2 (46.4)	41.2 (22.2, 60.3)	0.72	<0.001
RNFL	50.8 (29.7)	99.5 (32.7)	48.7 (35.8, 61.6)	1.26	<0.001
Macula	65.1 (28.2)	57.7 (22.2)	−7.4 (−18.5, 3.6)	−0.22	0.180

Values are mean (SD) in seconds; paired differences are Spectralis OCT2 minus Cirrus 6000; p-values from paired t-tests.

**Table 4:** Scan time comparisons in eyes with visual acuity worse than 20/40.

Measurement	Cirrus 6000 mean (SD)	Spectralis OCT2 mean (SD)	Mean difference (95% CI)	Cohen’s dz	P-value
Total	146.8 (24.7)	189.0 (46.2)	42.2 (−35.8, 120.2)	0.67	0.361
RNFL	55.4 (32.4)	116.2 (44.8)	60.8 (−25.2, 146.8)	0.88	0.313
Macula	91.4 (22.8)	72.8 (14.0)	−18.6 (−41.9, 4.7)	−0.99	0.188

Exploratory 5-patient subgroup. Values are mean (SD) in seconds; paired differences are Spectralis OCT2 minus Cirrus 6000; p-values from exact Wilcoxon signed-rank tests.

All 72 eyes included could complete both Spectralis OCT2 scans with an image quality score of 27.32 ± 3.48 for RNFL and 29.40 ± 4.35 for macula analysis. The image quality on the Spectralis OCT2 platform is expressed by the Q score, which is measured on a scale from 0-40; scores of at least 20 are considered acceptable (15). None of the scans required repeats due to low quality. For Cirrus 6000 scans, all 72 eyes completed both RNFL and macula imaging. The average image quality score was 8.41 ± 1.07 for RNFL and 8.85 ± 1.24 for macula. The image quality on the Cirrus 6000 is expressed by signal strength, which ranges from 0-10; scores of at least 6 are considered acceptable (16). Six patients required two repeat scans after their initial RNFL imaging in one eye due to low quality, and of those, two patients were not able to obtain an image quality score. These patients had ocular comorbidities of cataract (2), dry eye, corneal scar, cystoid macular edema, and posterior capsular opacification in the affected eye. The platforms use different vendor scales for image quality, so cross-device comparisons are descriptive only.

## Discussion

This investigator-driven study quantified time differences between two commonly used OCT devices to provide objective data that may inform workflow design, and resource allocation in glaucoma imaging. The Cirrus 6000 demonstrated significantly faster OCT acquisition times compared to the Spectralis OCT2, reducing total imaging time by 41 seconds per person. However, time savings are only realized if scans are successful on the first attempt. The Spectralis OCT2 scans were always successful and did not require any repeated attempts while the Cirrus 6000 sometimes failed to obtain an image due to poor quality. In high volume glaucoma practices, these differences have meaningful clinical implications, as the balance between acquisition speed and reliability may directly influence clinic efficiency, patient chair time, and overall workflow optimization.

The observed differences in acquisition time likely reflect both hardware and operational factors. Physicians and operators need to be aware that the higher the capture rate, the less light is transmitted, and any transmission interference from opacities is greater. For patients who are not dilated, a higher kHz may serve against better speed, depending on the clarity of their own media. In this study, the higher frequency light source employed by Cirrus 6000 enabled more rapid, higher resolution scans but also resulted in greater signal attenuation, particularly in the presence of media opacities and inadequate dilation (17–19). Operator experience further influences performance, as reflected by the superior performance of the Spectralis OCT2 in patients with visually significant media opacities, where it successfully acquired adequate scans in cases where the Cirrus 6000 did not, even with experienced technicians.

At the start of this trial, the SHIFT edition of the Spectralis OCT2 and the Cirrus 6000 were not cleared by the FDA. As such, this study strictly compared the 100 kHz Cirrus 6000 with the 85 kHz Spectralis OCT2. Since our study completed data collection, both the Cirrus 6000 and Spectralis SHIFT technology for OCT Angiography (OCTA) have received clearance from the FDA. SHIFT technology allows for scan speeds of up to 125 kHz, and an additional application is pending for speeds up to 250 kHz. However, standard OCT using SHIFT functionality is not yet authorized in the US. In markets where Spectralis SHIFT has been approved and is resourced, the higher kHz rates inherently increase the speed of capture given a state of sufficiently clear media. In addition to the Spectralis and Cirrus platforms, other newer automated devices have entered the market and may have faster acquisition speed.

When selecting an imaging platform, additional considerations such as cost, image quality, and progression analysis capabilities remain critical. There is no universally superior OCT system; rather, the optimal choice depends on the specific needs and characteristics of the clinical setting. For established patient populations with clear ocular media, Cirrus 6000 offers the advantages of speed and automation, which is an asset in clinics with high staff turnover or high patient volumes. In contrast, Spectralis OCT2, though less automated and slightly slower to set up, may be advantageous in practices with lower volume, stable staffing, and a patient population with significant comorbidities or media opacities. In such settings, the ability of the Spectralis OCT2 platform to acquire reliable scans despite challenging ocular media may outweigh the longer acquisition time. However, in a separate study comparing Spectralis OCT1 and OCT2 technology, a small number of incomplete scans occurred in patients with advanced glaucoma and optic disc abnormalities (17).

This study has several limitations that should be acknowledged. First, the comparison of acquisition times was conducted under real-world clinical conditions, which introduces variability related to operator experience, patient cooperation, and clinic workflow. Although this reflects typical practice settings, it may limit the ability to control for all confounding factors. We attempted to control this by matching technician skill level, randomizing which scan the patients received, and performing the scans consecutively. Second, media opacity severity was not formally graded, which may have influenced the observed differences in scan acquisition success between devices (20,21). The lack of a standardized grading system prevents a definitive subgroup analysis on exactly when the Cirrus 6000 begins to fail relative to the Spectralis OCT2. Additionally, only acquisition time was analyzed; other important parameters such as image quality, segmentation accuracy, and longitudinal progression analysis were not evaluated and may influence device selection in practice. Next, the findings may not be generalizable to all practice setting. Patient demographics and disease severity, technician expertise, and clinic volume can vary widely. As a single tertiary center, our population may differ from community-based or mixed subspecialty clinics. Because each device was operated by a different technician, differences in acquisition time could partly reflect technician-specific efficiency rather than device characteristics alone, representing a limitation of the study design. Finally, the assumptions in our sample size calculation assumptions did not match the results of the study. As a result, in some comparisons, our study may have been underpowered but offers insight into larger future studies.

In summary, the Cirrus 6000 demonstrated significantly faster OCT acquisition times compared to the Spectralis OCT2 module, offering meaningful efficiency gains in high-volume glaucoma clinics. However, its performance is more affected by media opacities, where the Heidelberg platform proved more adaptable. Device selection should therefore be individualized, considering clinic workflow, patient population, staff expertise, cost, and imaging priorities. Ultimately, the optimal choice of platform depends on aligning device strengths with the specific clinical environment to maximize imaging efficiency and diagnostic utility.

## Data Availability

The raw data supporting the conclusions of this article will be made available by the authors, without undue reservation.
